# P308: Degree of knowledge about transmission, prevention and risk perception of viral hepatitis b and c in manicures and pedicures in brazil

**DOI:** 10.1186/2047-2994-2-S1-P308

**Published:** 2013-06-20

**Authors:** ACDS Oliveira, S Scota, M costa, AM costa e Silva

**Affiliations:** 1 Educação Continuada e CCIH, Instituto de Infectologia Emílio Ribas, São Paulo, Brazil

## Introduction

Viral hepatitis is often transmitted by contaminated blood and sharp instrumental collective use, such as may occur with the removal of cuticles of the nails in salons. The manicures and pedicures remove their own cuticles, becoming a gateway to infectious agents during his career, come in contact with blood from customers, frequent occurrence in the removal of cuticles that may contaminate them, in addition to spreading disease to their customers. The scale of the problem in the area of beauty is apparent in Brazil for third place among global consumers of beauty products and the lack of sanitary control and biosecurity in these establishments.

## Objectives

To evaluate the level of information that the manicures / pedicures have about transmission routes and prevention of viral hepatitis B and C, and analyze the degree of perceived risk for accidental exposure to infectious agents by these professionals.

## Method

A prospective population-based epidemiological 100 manicures / pedicures done at salons and shopping malls central and peripheral neighborhoods, by random drawing, with a representative sample of the universe studied in São Paulo, conducted between November 2006 and February 2007. A questionnaire relevant to the goals.

## Results

In relation to the degree of knowledge, 72% of manicures and / or pedicures unaware routes HBV infection and 85% of HCV. Regarding prevention 93% did not know whether to prevent HBV and 95% of HCV. Regarding risk perception 54% reported not taking any prophylactic measure to come into contact with blood, while 46% took misconduct.

## Conclusion

The study suggests high potential risk procedures in manicures and pedicures in São Paulo, lack of care biosecurity, low perception of risk procedures, and inadequate sanitary control in salons in the city of São Paulo.

## Disclosure of interest

None declared.

